# Transradial Intracranial Aneurysm Coiling in a Seven-Year-Old Patient

**DOI:** 10.7759/cureus.24696

**Published:** 2022-05-03

**Authors:** Vamsi Krishna Gorijala, Gopi Krishna Vutla, Sudheer Chakravarthi, Anusha Guntamukkala, Kalyan Chakravarthy Sajja

**Affiliations:** 1 Neurology, Guntur Medical College, Guntur, IND; 2 Neurological Surgery, Government Medical College (Rajiv Gandhi Institute of Medical Sciences), Ongole, IND; 3 Interventional Neurology, Latha Super Speciality Hospital, Vijayawada, IND; 4 Interventional Neurology, Life Hospital, Guntur, IND; 5 Endovascular Neurosurgery, Thomas Jefferson University, Philadelphia, USA

**Keywords:** pediatric aneurysm, right radial artery access, transradial approach, coiling of the ruptured aneurysm, aneurysm coiling, endovascular aneurysm repair

## Abstract

Pediatric intracranial aneurysms (PIA) are very rare and can be fatal if left untreated. There are many treatment strategies including microsurgical and endovascular techniques. We feel that endovascular treatment using trans-radial access (TRA) is safe and convenient for PIA compared to the trans-femoral access (TFA), which is commonly employed in this population. We present the case of the youngest patient in the world whose ruptured aneurysm was treated with endovascular coiling via the TRA. The seven-year-old patient was brought to the ER with a severe headache. He had several episodes of vomiting and an episode of seizure as well. Computerized tomography (CT) of the brain showed subarachnoid hemorrhage. A magnetic resonance angiogram (MRA) showed an aneurysm at the bifurcation of the right internal carotid artery (ICA). An intermediate catheter/microcatheter system was used to navigate up into the ICA and then into the aneurysm. Two coils were deployed with good packing. The patient had a good clinical recovery and is currently doing good without any neurological deficits. With the availability of newer devices, we believe the TRA will be widely used in the coming years. We need to have larger randomized controlled trials to really understand the advantages of TRA in this patient population.

## Introduction

Pediatric intracranial aneurysms (PIAs) in patients ≤ 18 years old are rare and their pathophysiology is poorly understood [1]. They have a male predominance and are usually found at sites of arterial bifurcation, most commonly at the internal carotid artery (ICA) bifurcation [1]. They can rupture leading to subarachnoid hemorrhage (SAH). The most common presenting complaints are sudden severe headaches, loss of consciousness, focal neurological deficits, seizures, and direct compressive effects [1]. A PIA needs to be treated immediately due to the inherent risks of aneurysm growth, rupture, etc. PIAs are treated with open approaches like microsurgical clipping or endovascular approaches. The treatment of ruptured aneurysms has been moving towards an endovascular approach as it offers better clinical trends [1]. Most operators use the trans-femoral approach (TFA) to operate on PIAs. There is increasing evidence in recent times that the trans-radial approach (TRA) has lower access site complications and good surgical outcomes [2]. We present a case of endovascular coiling of a ruptured intracranial aneurysm via the TRA in a seven-year-old child, which to our knowledge is the youngest patient to be treated with coiling via the TRA.

## Case presentation

The patient, who is seven years old with no prior medical history, presented to the ER with a severe headache for five days. The headache was relieved with medication. The patient also had multiple episodes of emesis and a seizure after which the parents brought the patient to the ER. Glasgow Coma Scale (GCS) at presentation was 15 (E_4_V_5_M_6_). There was no prior history of seizures or other risk factors for seizures.

Complete blood counts and complete metabolic profile were all within normal limits. Echocardiography and ECG were normal. CT brain showed subarachnoid hemorrhage. An MRI and a magnetic resonance angiogram (MRA) were obtained, which showed subarachnoid hemorrhage and a 4 x 3.1 mm right A1-A2 aneurysm.

**Figure 1:**
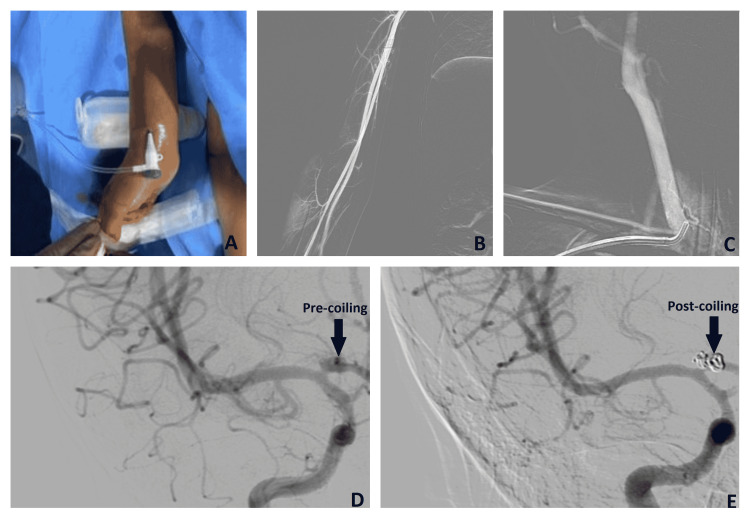
(A) Procedure performed via the trans-radial access (TRA); (B) Assessing the right radial artery; (C) Right carotid artery is accessed via the right radial artery; (D) Cerebral angiogram showing the aneurysm before coiling (arrow); (E) Postoperative angiogram showing aneurysm after coiling (arrow).

After a discussion of available options with the parents, a decision was made for endovascular coiling. The right radial artery was accessed (Figure 1A) with a Prelude® 6F radial sheath (Merit Medical Systems, Inc., Utah, United States) (Figure 1B). An ASAHI FUBUKI® 6F guide catheter (Asahi Intecc USA, Inc., Tustin, California, United States) over a Tempo™ Angiographic Catheter Berenstein II 5 Fr (Cordis Corporation, Hialeah, Florida, United States) and 0.035 Glidewire® (Terumo Corporation, Shibuya City, Tokyo, Japan) were used to access the right carotid artery (Figure 1C). The 6F guide catheter was taken up into the ICA over roadmap guidance. Next, a DAC® 038 Distal Access Catheter (Stryker Corporation, Kalamazoo, Michigan, United States) and a Frepass® Microcatheter 10 (Lepu Medical Technology, China) 150 cm over a Synchro®-14 Neuro Guidewire (0.014) (Stryker Corporation, Kalamazoo, Michigan, United States) were used to access the aneurysm (Figure 1D). Perdenser® Embolic Coil System 3D 3.5cm x 8 cm, and 2D 1.5 x 3cm coils (Lepu Medical Technology, China) were deployed (Figure 1E). Check angiogram showed no filling of the aneurysm. The microcatheter and the intermediate catheters were removed. The guide catheter and the sheath were then removed and the access site was secured with manual compression. The patient had a good clinical recovery and is currently without any neurological deficits. A follow-up angiogram is planned for six months from the date of coiling.

## Discussion

PIAs are rare but can be fatal. They are more prone to rupture compared to adults, leading to SAH [3]. The threshold to treat intracerebral aneurysms is, therefore, lower in the pediatric population. These aneurysms can be managed with surgery including clipping, reconstruction, and bypassing of the parent vessel or endovascular approach - coiling, stent-assisted coiling, balloon-assisted coiling, flow diversion, and intravascular devices [3]. 

Sanai et al. described the effectiveness and durability of microsurgical procedures in completely eliminating the aneurysm compared to the endovascular approach [4]. There were no recurrences with microsurgical methods. But the main concern is slightly increased mortality and morbidity when compared to the endovascular approach [4].

Larger randomized controlled trials like the International Subarachnoid Aneurysm Trial (ISAT) showed that endovascular coiling is better for ruptured aneurysms as it is associated with better outcomes in terms of disability-free survival [5]. Endovascular procedures are minimally invasive and offer easy peripheral access and fewer complications. But the rates of recurrence can be higher [5]. 

Conventionally, neuroendovascular procedures were done using the TFA. But recent studies established the safety and effectiveness of the TRA for these procedures [6]. They offer shorter hospital stays, greater patient satisfaction, and fewer complications [6]. The TRA for the treatment of PIA is not very prevalent. A study conducted by Goland et al. established the safety and feasibility of the TRA for PIA treatment [7]. The youngest patient in their series was 15 years old. Fadi et al. have performed the TRA approach in younger patients for intraarterial chemotherapy for retinoblastoma [8]. We are describing this procedure for aneurysm coiling in a patient who is seven years old, which effectively makes him the youngest patient in the world to receive endovascular aneurysm coiling via the TRA approach. 

TRA for intracranial aneurysm coiling is gaining popularity with the advent of better devices. We need more experience to really know the advantage of the TRA versus the TFA in this demographic.

## Conclusions

We feel that coiling via the TRA might be easier in the pediatric population compared to the TFA. There are fewer access-site and other complications, and good clinical outcomes. Hospital stay is also minimized. With the availability of better devices and good operator compliance, the TRA will gain better reception in the future. Children cannot describe their complaints like adults. Hence, we believe that the healthcare providers must be alert and maintain a low threshold for imaging to diagnose aneurysms in this population. We need to perform large-scale randomized controlled trials comparing the TRA versus the TFA for aneurysm coiling to know the benefit. We believe this is just the beginning and more operators will use the TRA for aneurysm coiling in the coming years. 
